# Extreme-Depth Re-sequencing of Mitochondrial DNA Finds No Evidence of Paternal Transmission in Humans

**DOI:** 10.1371/journal.pgen.1005040

**Published:** 2015-05-14

**Authors:** Angela Pyle, Gavin Hudson, Ian J. Wilson, Jonathan Coxhead, Tania Smertenko, Mary Herbert, Mauro Santibanez-Koref, Patrick F. Chinnery

**Affiliations:** 1 Wellcome Trust Centre for Mitochondrial Research, Newcastle University, Newcastle-upon-Tyne, United Kingdom; 2 Institute of Genetic Medicine, Newcastle University, Newcastle-upon-Tyne, United Kingdom; Max Planck Institute for Biology of Ageing, GERMANY

## Abstract

Recent reports have questioned the accepted dogma that mammalian mitochondrial DNA (mtDNA) is strictly maternally inherited. In humans, the argument hinges on detecting a signature of inter-molecular recombination in mtDNA sequences sampled at the population level, inferring a paternal source for the mixed haplotypes. However, interpreting these data is fraught with difficulty, and direct experimental evidence is lacking. Using extreme-high depth mtDNA re-sequencing up to ~1.2 million-fold coverage, we find no evidence that paternal mtDNA haplotypes are transmitted to offspring in humans, thus excluding a simple dilution mechanism for uniparental transmission of mtDNA present in all healthy individuals. Our findings indicate that an active mechanism eliminates paternal mtDNA which likely acts at the molecular level.

## Author Summary

Emerging evidence raises the possibility that human mitochondrial DNA (mtDNA) is not strictly maternally inherited, but it has not been technically possible to test this hypothesis directly. We identified trios with discordant mtDNA haplotypes, parent-offspring trios were validated using polymorphic microsatellites, and then used extreme-high depth mtDNA re-sequencing to look for paternally transmitted mtDNA. Despite having up to ~1.2 million-fold coverage of mtDNA, we find no evidence that paternal mtDNA haplotypes are transmitted to offspring in humans. Our findings exclude a simple dilution mechanism for uniparental transmission of mtDNA present in all healthy individuals.

## Introduction

In eukaryotes, cytoplasmic genes are generally inherited from the mother. The mechanisms responsible for this appear to differ between organisms. The elimination of mitochondrial DNA (mtDNA) during spermatogenesis prevents paternal transmission in *Drosophila melanogaster* [1]. Conversely, in the Japanese medaka fish *Oryzias latipes*, sperm mtDNA is lost after fertilization [2]. In cows and humans, sperm mtDNA is eliminated from two or four cell embryos [3,4], and sperm loss may also occur throughout embryogenesis in mice [5].

Although sperm mitochondria are tagged with ubiquitin and actively destroyed through P62 and LC3-mediated autophagy [6,7], there is no direct evidence showing the destruction of sperm mitochondrial DNA (mtDNA) [8], and there are several examples where paternal mtDNA has escaped this process. Extensive paternal transmission of mtDNA has been observed in the marine mussel (*Mytillus sp*) [9], and occasionally in the fruit fly (*Drosophila melanogaster*)[10], *Lepidopteran* insects and the honey bee (*Apis mellifera*) [11]. For the most part, the “leakage” of paternal mtDNA during transmission in mammals has only been observed in atypical situations such as inter-strain breeding in mice (*Mus musculus*) [12], or following *in vitro* embryo manipulation in cattle (*Bos taurus*) [13]. However, the description of a paternally-derived 2 base pair pathogenic deletion in the mtDNA *MTDN2* in a 28-year-old man with a mitochondrial myopathy [14], the persistence of human sperm-derived mtDNA when introduced into somatic cells [15] and in abnormal fertilised human embryos [16], coupled with evidence of paternally transmitted mtDNA in healthy sheep (*Ovis aries*) [17], and the great tit (*Parus major*) in its natural habitat [18], questions the accepted dogma of exclusive maternal transmission.

In humans, the debate hinges on the analysis of mtDNA sequences at the population level. By studying partial or complete mtDNA sequences from individuals across the globe, some have argued that the co-occurrence of phylogenetically unrelated genetic variants indicates inter-molecular recombination between paternal and maternal mitochondrial genomes [19], but others have argued that the high mtDNA mutation rate confounds this analysis through the generation of homoplasy [20], which can reach ~20%. Recent experimental data showed no evidence of the active removal of sperm mtDNA from developing mammalian embryos [8], pointing towards a passive dilution process based on differences in the amount of mtDNA between human gametes. Here we test this hypothesis directly.

## Results

First we determined the proportion of paternal haplotypes transmitted to the offspring assuming no preferential destruction of sperm-derived mtDNA. We measured the amount of mtDNA in healthy human sperm and pre-fertilization oocytes on the same assay plate. This gave a mean ratio of 1:15,860, in keeping with previous reports [8,21–23]. Based on these observations, the 95% prediction interval for the proportion of paternal haplotypes at fertilization is 10^–5^ to 1.8x10^-4^.

**Fig 1:**
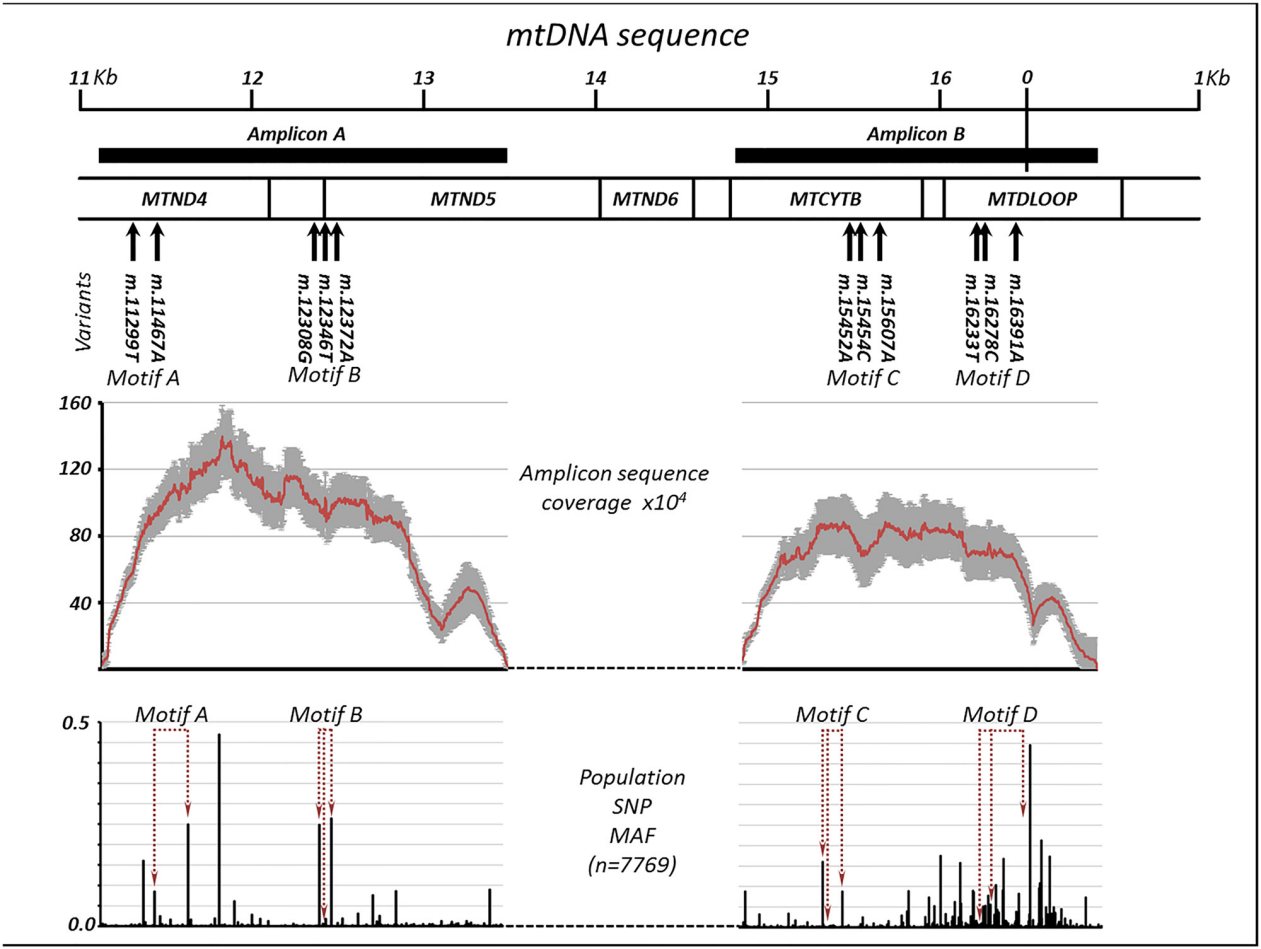
Extreme deep sequencing in trios with discordant paternal and maternal mitochondrial DNA. (a) Position of the discordant haplotypes on the mitochondrial genome. Thick horizontal bars show the position of the PCR amplicons. (b) Average sequencing depth +/- 95% confidence intervals for the two amplicons in all four trios. (c) Population variant allele frequencies for the two amplicons, indicating position of motif variants.

Next we developed an extreme-depth mtDNA re-sequencing assay to detect very low levels of paternal haplotypes. Given previous work showing a background level of ~1% heteroplasmy for single variants using deep mtDNA re-sequencing [24], we set out to identify trios where the father and the child had two or more variant differences within a <200bp stretch of mtDNA, thus allowing the detection of extremely rare paternal haplotypes at a much lower heteroplasmy level captured within the same sequencing read. Sanger sequencing of the mtDNA from 99 mother-father-child trios from the Avon Longitudinal Survey of Parents and Children identified four different trios with >2 discordant alleles (subsequently referred to as discordant variants, S1 & S2 Tables, Fig 1A & S1 Fig). Parent-offspring trios were confirmed with >99.9% accuracy using 16 short microsatellites in all members of each trio. Next we designed PCR amplicons spanning the four discordant regions. Each amplicon was >2Kb to eliminate nuclear pseudogene amplification, and BLAST analysis predicted exclusive annealing to mtDNA (Fig 1A), which was confirmed by the failure to generate a template from rho-0 cells devoid of mtDNA. All four amplicons were amplified in all four trios using ultra-high fidelity polymerase (PrimeSTAR GXL, TaKaRa Bio Europe, fidelity = 1 error/~16,230 base pairs), allowing each trio to act as a control for the others. Extreme high-depth re-sequencing (Illumina MiSeq, 250bp paired-end reads) yielded stable coverage spanning the region of interest in the offspring (Fig 1B).

We then compared the frequency of minor alleles and haplotypes both within and between the four trios. The frequency of isolated single variants was similar to that observed previously at lower depth at ~ 0.5% [24]. As expected, the number of minor haplotypes containing two or three variants was substantially less (Table 1). Overall, the frequency of minor haplotypes containing two variants approximated the square root of the single variant frequency, and the frequency of the three-allele haplotypes was approximately equal to the cube root of the single variant frequency. These observations were in keeping with a random background mutation frequency affecting single base-pairs of ~0.5% [24]. The observed alleles contributing to the ultra-rare haplotypes did not correspond to commonly co-occurring population variants, making external sample contamination unlikely (Fig 1D and S2 Table). The frequency of unexpected maternal or paternal haplotypes was greater in other members of the same family trio than in other trios. Given that all of the trios were analysed in the laboratory simultaneously, these rare shared haplotypes probably arose through very low-level contamination at the time the original samples were taken, in the order of <10^–5^ molecules (Table 1). We incorporated these observations in a significance test of our findings, and determined whether we had adequate statistical power to detect paternally inherited mtDNA.

**Table 1:** Frequency of rare haplotypes in trios with discordant paternal and maternal mitochondrial DNA.

					Trio 1—Motif A		Trio 2—Motif B			Trio 3—Motif C		Trio 4—Motif D	
							*m*.*12308A*	*m*.*12308G*		*m*.*15452G*	*m*.*15452A*	*m*.*16233T*	*m*.*16233C*
					*m*.*11299T*	*m*.*11299C*	*m*.*12346C*	*m*.*12346T*		*m*.*15454T*	*m*.*15454C*	*m*.*16278C*	*m*.*16278T*
					*m*.*11467A*	*m*.*11467G*	*m*.*12372G*	*m*.*12372A*		*m*.*15607G*	*m*.*15607A*	*m*.*16391A*	*m*.*16391G*
***ID***	***Family***	***Relationship***	***Exp*. *Motif***	***Aligned Reads***	***Maternal***	***Paternal***	***Maternal***	***Paternal***	***Aligned Reads***	***Maternal***	***Paternal***	***Maternal***	***Paternal***
**S1**	**Trio 1**	**Mother**		8478213	**252906**	**11**	411706	1	5397958	0	80	53821	0
**S2**		**Father**	**Motif A**	10785620	**27**	**323903**	45	318	3516279	0	67	2	0
**S3**		**Child**		10872601	**322675**	**6**	520938	1	5990929	0	85	60397	0
**S4**	**Trio 2**	**Mother**		8459438	237212	1	**412320**	**25**	14010367	0	153	12	162
**S5**		**Father**	**Motif B**	7981400	102	2088	**19**	**326148**	5930021	0	86	0	0
**S6**		**Child**		8184182	234586	2	**343282**	**16**	4096769	0	77	0	52
**S7**	**Trio 3**	**Mother**		9500936	237181	1	414430	0	5571846	**86457**	**5**	0	1
**S8**		**Father**	**Motif C**	8124010	183	5986	118	115	6293236	**0**	**162098**	0	0
**S9**		**Child**		7307511	212099	2	385255	0	9868902	**71994**	**4**	0	0
**S10**	**Trio 4**	**Mother**		10151535	254517	1	468255	0	3158406	0	38	**26346**	**2**
**S11**		**Father**	**Motif D**	8416736	238561	2	402764	0	7067065	0	94	**23**	**76244**
**S12**		**Child**		8141655	235512	3	469861	0	8799863	1	145	**78760**	**4**

Two or three-allele mtDNA motifs define the discordant haplotypes also shown in Fig 1A. Raw reads from extreme high-depth mtDNA sequencing across all four motifs to detect paternal haplotypes. The number of possible paternal ‘reads’ in the children was the same as the mothers from the same trio, in keeping with background noise.

**Fig 2:**
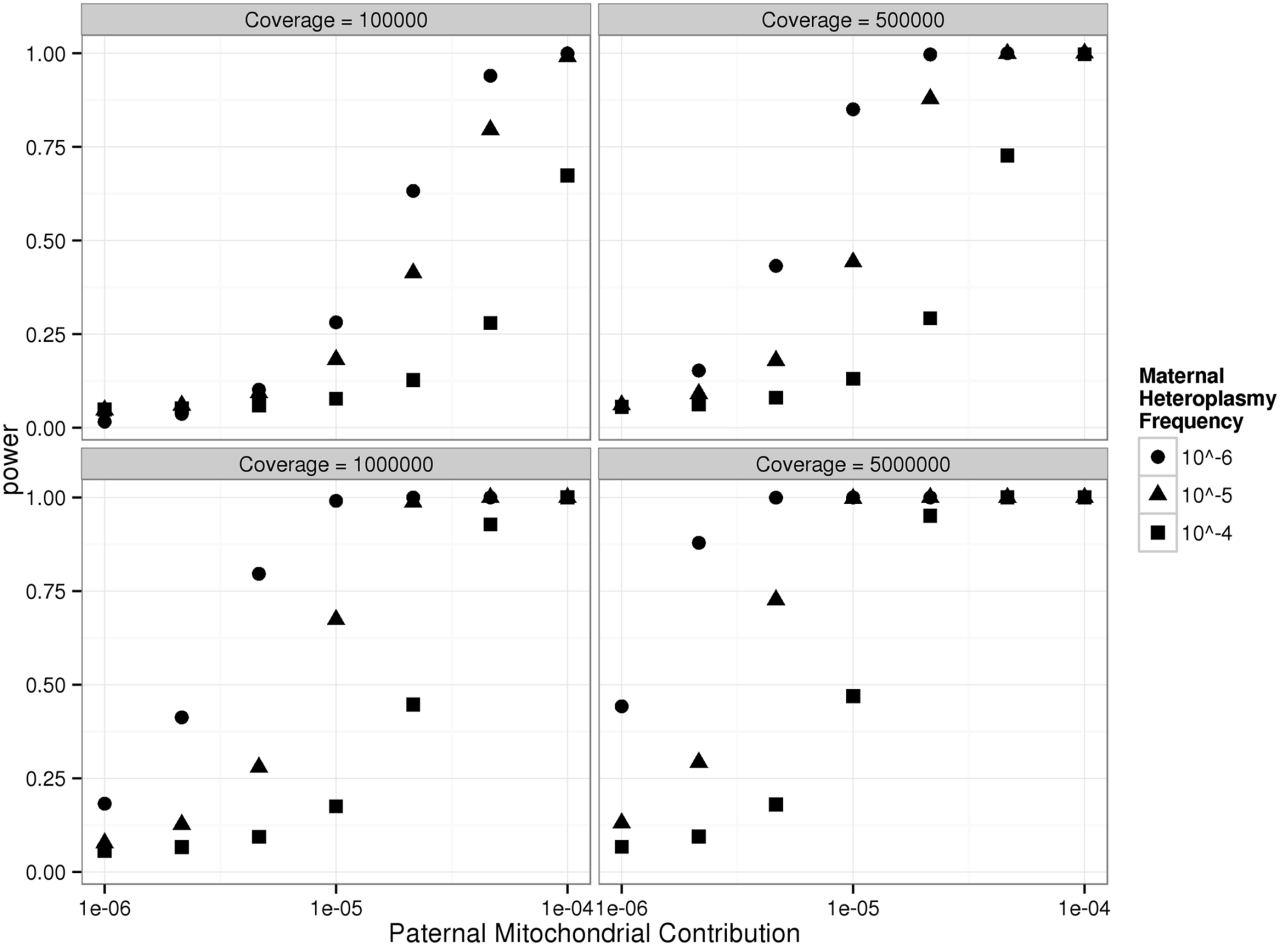
Power to detect paternally transmitted mtDNA in the children studied here based on the observed ultra-deep mtDNA sequence data. Calculations are based on the comparison of two Poisson distributions as described by Skellam [25], assuming the same mtDNA fold coverage in mothers and offspring (see text for a full description of the methods). Each graph shows the power to detect a paternal contribution to the mtDNA in the offspring of the trios studied here based for different degrees of ultra-deep sequencing coverage, and for different background levels of mtDNA heteroplasmy seen in the mothers, which in this study were 4.3 x 10^–5^, 6.0 x 10^–5^, 5.7 x 10^–5^, and 7.6 x 10^–5^.

The power to detect paternally inherited mtDNA was estimated by determining the difference between mismatched haplotype frequencies in mothers and children. These are simply modelled by Poisson distributions with a without paternal contributions, and the difference is described by a Skellam distribution [25]. The power was investigated for a range of theoretical paternal mtDNA contributions, and the observed background heteroplasmy levels in mothers (Fig 2). For the observed background heteroplasmy levels in the mothers (4.3 x 10^–5^, 6.0 x 10^–5^, 5.7 x 10^–5^, and 7.6 x 10^–5^, Table 1), sequencing at >300,000-fold depth in the relevant offspring had >80% power to detect the predicted level of transmitted paternal mtDNA.

A bootstrap test was used to determine whether the observed discordant haplotypes in the children were consistent with the predicted paternal contribution, incorporating the 95% confidence intervals for proportion of sperm haplotypes in the oocytes (10^–5^ to 1.8x10^-4^). A paternal contribution was not compatible for trios A and B (p = 0.004, p = 0.016), but there was insufficient evidence to reject the other two trios (p = 0.21, p = 0.12) that had a lower total coverage for the full range of possible transmitted levels of sperm mtDNA. The power calculation suggests that the uncertainty in two trios likely reflects the confidence interval for our estimate of sperm haplotypes in the fertilized oocyte, and not directly reflect the likelihood of any paternal transmission of mtDNA.

The full data set and relevant code is available from the authors and at: https://www.staff.ncl.ac.uk/i.j.wilson/PaternalTransmission

## Discussion

When taken together these findings indicate that the extremely rare variant haplotypes seen in the offspring are highly unlikely to have arisen through the passive ‘leakage’ of paternal mtDNA within the trio. Lower levels of paternal mtDNA transmission are also unlikely because we did not see common mtDNA population haplotypes in any of the individuals studied (Fig 1D), which would be expected if there were frequent paternal leakage in the human population. The lack of common population haplotypes at extreme high depth also shows that our experimental approach was not subject to significant cross-contamination. Finally, we show that there is little to be gained by sequencing at >5000-fold if single variants are of interest, because the ‘noise’ level will prevent further resolution of low-level heteroplasmy.

The ‘background noise’ level that we observed has several potential origins. First, the near exponential decrease in frequency of single variants, two variant, and three variant haplotypes is consistent with a random background mutation frequency introduced by DNA replication errors, either within the biological system or through PCR amplification. We did not observe patterns of nucleotide changes that would imply a direct insult to the DNA templates (such as C>U deamination damage). Finally, even at extreme high depth (>1 million reads in some trios) we saw negligible or no haplotypes observed in other trios. Given that the trios were analysed together, this means that our laboratory procedures were robust with negligible cross contamination. It is therefore likely that the rare paternal haplotypes seen in maternal samples (Table 1) were introduced when the samples were collected.

Our observations were made on DNA extracted from buccal swabs. It is thus theoretically possible that paternal mtDNA was originally transmitted to the offspring and subsequently lost from buccal cells before the DNA samples were acquired. The loss of mutated mtDNA has been observed in patients with mtDNA diseases, most typically m.3243A>G, probably through selection against a deleterious allele at the stem cell level [26]. However, for most inherited heteroplasmic mtDNA mutations (eg m.8993T>C), the percentage level of the mutation is the same in a wide range of different tissues [27], including buccal swabs. It is therefore likely that if there were paternal transmission, this would be detected in all tissues. Moreover, since variants we studied here are haplogroup markers and are unlikely to have significant biochemical consequences [28], it is highly unlikely that a paternal haplotype allele would be lost from a particular tissue through selection. Absolute reassurance would be provided by studying other tissues, but this approach has its own difficulties because somatic point mutations accumulate in non-dividing tissues, increasing the background ‘noise’ levels to a point that would preclude the detection of passively transmitted paternal mtDNA.

Given that human paternal mtDNA can be detected in very early embryos [4], what is the mechanism underpinning exclusive maternal inheritance of mtDNA in humans? Recent observations that ubiquitination and P63/LC3 tagging of sperm mitochondria does not lead to mitophagy in mice, the mechanism is likely to act at the level of the mitochondrial genome [8]. This is plausible, given recent observations in heteroplasmic mice where a similar mechanism was proposed for heteroplasmy segregation during inheritance [29,30], and within different tissues and organs during life [31], where selection against specific mtDNA molecules occurs at levels well below the level required to cause a biochemical defect affecting oxidative phosphorylation within the cell. A similar mechanism was proposed following the introduction of sperm mtDNA into somatic cells [15]. Although it remains to be determined how this selection process occurs at the molecular level, it is of fundamental importance in preventing the accumulation of deleterious mutations in the human population, effectively taking Muller’s ratchet [32] ‘down a gear’.

## Materials and Methods

### Ethics statement

Ethical approval for the study was obtained from the ALSPAC Ethics and Law Committee and the Local Research Ethics Committees. The ALSPAC study web site contains details of all the data that is available on the cohort through a fully searchable dictionary (http:www.bris.ac.uk/alspac/researchers/data-access/data-dictionary/).

### Quantification of mtDNA in sperm and oocytes

Excess oocytes were collected after follicular reduction from healthy donors, lysed for 16 hours in 50mM Tris-HCl, pH 8.5, with 0.5% Tween 20 and 200ng/ml proteinase K, at 55°C, followed by heat inactivation at 95°C for 10 minutes. Three single oocytes and 43 sperm DNA samples were analysed using quantitative real-time PCR (qPCR). A multiplex qPCR assay, using probes and primers targeting a region of *MT-ND1* and the nuclear housekeeping gene β-2 microglobulin (*β2M)*, were used to measure mtDNA copy number on a CFX96 Touch™ Real-Time PCR Detection System (BioRad, USA).

### Identification of mtDNA discordant trios

Buccal DNA samples were extracted in a different laboratory from 100 mother-father-child trios obtained from the Avon Longitudinal Study of Parents and Children Cohort (ALSPAC) [33,34]. Mitochondrial DNA (mtDNA) haplogroup defining single nucleotide polymorphisms (SNPs, www.phylotree.org) were determined in each mother and father by Sanger sequencing specific regions of the mitochondrial DNA genome. (Big Dye v3.1 kit and capillary electrophoresed on an ABI3130xl Genetic Analyzer, Life Technologies, Warrington, UK). Alignment and variant calling was performed using SeqScape software (v2.1.1, Applied Biosystems) reference to the GenBank sequence NC_012920.1. Four trios were identified with >2 discordant variants falling within a ~250 bp read-length (S1 Table). Parent-offspring trios were confirmed using the Promega Powerplex 16 HS system (Promega, Southampton, UK, performed by NorthGene Ltd.).

### mtDNA isolation and enrichment

2x 2Kb mtDNA amplicons were designed to span the discordant regions in each trio (Fig 1A) and to avoid nuclear pseudogene co-amplification with a high-fidelity polymerase (PrimeSTAR GXL DNA Polymerase, TaKaRa Bio Europe, France). Primer sequences were: set 1 = TATCCAGTGAACCACTATCAC-F (m.11010-11030) and GGGAGGTTGAAGTGAGAGG-R (m.13453-13435); set 2 = ATTCATCGACCTCCCCACC-F (m.14797-14815) and CTGGTTAGGCTGGTGTTAGG-R (m.389-370). Initially, primer efficiency and specificity was assessed as successful after no amplification of DNA from rho0 cell lines, which contain no mtDNA. Amplified products were assessed by gel electrophoresis against DNA+ve and DNA-ve controls, and quantified using a Qubit 2.0 fluorimeter (Life Technologies, Paisley, UK). Each amplicon was individually purified using Agencourt AMPure XP beads (Beckman-Coulter, USA), pooled in equimolar concentrations and re-quantified.

### Extreme depth mtDNA sequencing

Amplicons were ‘tagmented’, amplified, cleaned, normalised and pooled into a multiplex using the Illumina Nextera XT DNA sample preparation kit (Illumina, USA). Multiplex plex pools were sequenced using MiSeq Reagent Kit v3.0 (Illumina, USA) in paired-end, 250 bp reads on the same flow cell.

### Bioinformatic analysis

Post-run FASTQ files were analysed using an in-house developed bioinformatic pipeline. Reads were aligned to the rCRS (NC_012920) using BWA v0.7.10 [35], invoking—mem [35]. Aligned reads were sorted and indexed using Samtools v0.1.18 [36]. Variant calling was performed in tandem using VarScan v2.3.13 [37,38], (minimum depth = 1500, supporting reads = 10 and variant threshold = 1.0%) and LoFreq v0.6.1 [39]. Concordance calling between VarScan and LoFreq was >99.5%. Concordant variants were annotated using ANNOVAR v529 [40]. In-house Perl scripts were used to extract base/read quality data and coverage data.

Potential paternal haplotypes were identified from the pool of analysed reads (*.sam files) using command line scripting, generating motif-specific counts for each trio. For example, [grep—c 'CCTCACTGCCCAAGAACTATCAAACTCCTGAGCCAACAACTTAATATGACTAGCTTACACAATAGCTTTTATAGTAAAGATACCTCTTTACGGACTCCACTTATGACTCCCTAAAGCCCATGTCGAAGCCCCCATCGCTGGGTCAATAGTACTTGCCGCAGTACTCTTG\|CAAGAGTACTGCGGCAAGTACTATTGACCCAGCGATGGGGGCTTCGACATGGGCTTTAGGGAGTCATAAGTGGAGTCCGTAAAGAGGTATCTTTACTATAAAAGCTATTGTGTAAGCTAGTCATATTAAGTTGTTGGCTCAGGAGTTTGATAGTTCTTGGGCAGTGAGG'. /*.sam] was used to identify whole reads containing m.11299C and m.11467G in forward and complement orientations (corresponding bases underlined). Counts were generated for all possible permutations of motifs, in all available samples.

### Statistical analysis

The mtDNA counts for healthy human sperm had mean 77.2 (SD 53.9, n = 43) and oocytes had mean 1220000 (SD 183000, n = 3). An empirical distribution for the ratio of sperm to oocyte mtDNA levels immediately after fertilization was estimated by bootstrapping. 100,000 individual mtDNA counts were sampled from the raw sperm mtDNA counts and 10^5^ corresponding oocyte mtDNA counts were drawn from a normal distribution with mean and standard deviation as above for the oocytes. A 95% prediction interval was then calculated from the 2.5^th^ and 97.5^th^ percentiles of this ratio of sperm mtDNA to oocyte count.

A hypothesis test for paternal transmission was performed by bootstrapping under the null hypothesis that the discordant haplotypes were due to paternal inheritance at the ratio predicted from the direct measurements on individual gametes. For each of the four discordant haplotypes, 10^5^ sampled ratios were simulated in the same way as for the prediction interval, and from this 10^5^ replicate discordant child haplotypes were generated by binomial sampling, with ***p*** equal to these ratios and ***n*** equal to the total coverage over all haplotypes for this motif and trio. Observed background noise was added to this count at a Poisson rate equal to the discordant maternal haplotype rate. The simulated maternal count was pure Poisson noise, generated at the same rate as the child noise. The bootstrapped differences in proportion were then compared to the observed data, and extreme values are evidence to reject the null hypothesis. The p-value is estimated percentile rank of the observed data in the bootstrapped distribution.

## Supporting Information

S1 Fig Mitochondrial DNA haplotypes for the individuals in each trio studied. (TIFF)

S1 Table Mitochondrial DNA (mtDNA) haplogroup and mtDNA SNPs in the four trios showing discordant variants within a ~250bp stretch of mtDNA. M = mother, F = father, C = child.
(DOCX)

S2 Table Minor allele frequencies (MAF) for the discordant paternal haplotypes seen in 7769 population controls. The paternal haplotypes are exceptionally rare in the population and thus unlikely to have been introduced by contamination from other sources. Motif refers to the paternal haplotype specified in Fig 1A. SNP = single nucleotide polymorphism.
(DOCX)
