# Supplementary figures and images for: Extreme-Depth Re-sequencing of Mitochondrial DNA Finds No Evidence of Paternal Transmission in Humans
Source: PLoS Genet. 2015 May 14;11(5):e1005040. doi: 10.1371/journal.pgen.1005040 (PMC4431825; doi:10.1371/journal.pgen.1005040)

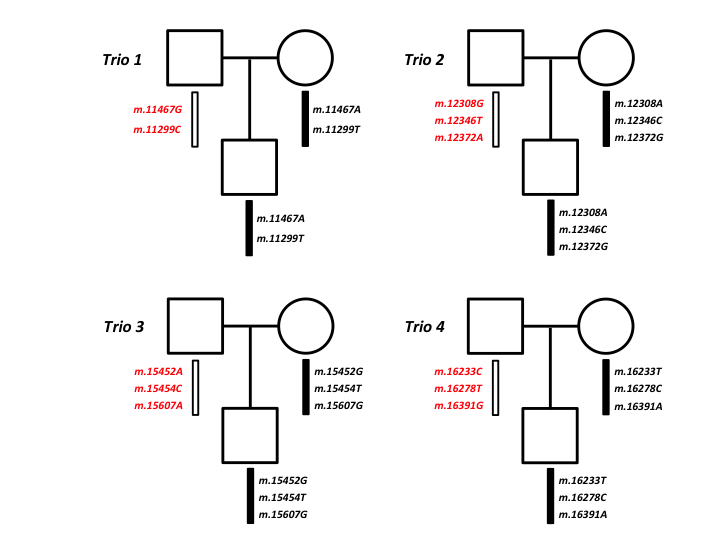

Supplement: S1 Fig — (TIFF) [file pgen.1005040.s001.tiff]
