# Supplementary material for: Extreme-Depth Re-sequencing of Mitochondrial DNA Finds No Evidence of Paternal Transmission in Humans
Source: PLoS Genet. 2015 May 14;11(5):e1005040. doi: 10.1371/journal.pgen.1005040 (PMC4431825; doi:10.1371/journal.pgen.1005040)
Supplement: S1 Table — M = mother, F = father, C = child. (DOCX) [file pgen.1005040.s002.docx]

***Table S1*** – Mitochondrial DNA (mtDNA) haplogroup and mtDNA SNPs in the four trios showing discordant variants within a ~250bp stretch of mtDNA. M=mother, F=father, C=child.

| ***Trio*** | ***Sample*** | ***mtDNA Haplogroup*** | ***Discordant Variants*** | | |
| --- | --- | --- | --- | --- | --- |
|  |  |  |  |  |  |
| 1 | M | U | m.11299T | 11467A |  |
|  | **F** | **X** | **m.11299C** | **11467G** |  |
|  | C | U | m.11299T | 11467A |  |
|  |  |  |  |  |  |
| 2 | M | K | m.12308A | m.12346T | m.12372A |
|  | F | I | **m.12308G** | **m.12346C** | **m.12372G** |
|  | C | K | m.12308A | m.12346T | m.12372A |
|  |  |  |  |  |  |
| 3 | M | T | m.15452A | m.15454T | m.15607G |
|  | **F** | **U** | **m.15452G** | **m.15454C** | **m.15607A** |
|  | C | T | m.15452A | m.15454T | m.15607G |
|  |  |  |  |  |  |
| 4 | M | I | m.16233T | m.16278C | m.16391A |
|  | **F** | **R** | **m.16233C** | **m.16278T** | **m.16391G** |
|  | C | I | m.16233T | m.16278C | m.16391A |
|  |  |  |  |  |  |
