# Supplementary material for: Extreme-Depth Re-sequencing of Mitochondrial DNA Finds No Evidence of Paternal Transmission in Humans
Source: PLoS Genet. 2015 May 14;11(5):e1005040. doi: 10.1371/journal.pgen.1005040 (PMC4431825; doi:10.1371/journal.pgen.1005040)
Supplement: S2 Table — The paternal haplotypes are exceptionally rare in the population and thus unlikely to have been introduced by contamination from other sources. Motif refers to the paternal haplotype specified in Fig 1A. SNP = single nucleotide polymorphism. (DOCX) [file pgen.1005040.s003.docx]

***Table S2*** – Minor allele frequencies (MAF) for the discordant paternal haplotypes seen in 7769 population controls. The paternal haplotypes are exceptionally rare in the population and thus unlikely to have been introduced by contamination from other sources. Motif refers to the paternal haplotype specified in Fig.1a. SNP=single nucleotide polymorphism.

| MOTIF | SNP | MAF (n=7769) |
| --- | --- | --- |
| A | m.11299C>T | 0.085 |
|  | m.11467G>A | 0.251 |
|  |  |  |
| B | m.12308A>G | 0.251 |
|  | ***m.12346C>T*** | ***0.006*** |
|  | m.12372G>A | 0.264 |
|  |  |  |
| C | m.15452G>A | 0.160 |
|  | ***m.15454T>C*** | ***0.007*** |
|  | m.15607G>A | 0.087 |
|  |  |  |
| D | ***m.16233C>T*** | ***0.007*** |
|  | m.16278T>C | 0.055 |
|  | ***m.16391G>A*** | ***0.009*** |
